# HBV-encoded circRNA-5 promotes hepatocellular carcinoma progression by regulating the miR-9-3p/Hippo signaling axis

**DOI:** 10.1128/spectrum.01439-25

**Published:** 2025-09-05

**Authors:** Yong Yang, Yongshi Shen, Yuan Dang, Jingyun Huang, Yinghua Su, Jialiang Zhang, Xin Jiang, Xinying Luo, Jianmin Wang, Jingfeng Liu

**Affiliations:** 1 Innovation Center for Cancer Research, Clinical Oncology School of Fujian Medical University, Fujian Cancer Hospital 66552 , Fuzhou, China; 2 Fujian Key Laboratory of Advanced Technology for Cancer Screening and Early Diagnosis, Fujian Cancer Hospital, Fuzhou, China; 3 Department of Intensive Care Unit, Clinical Oncology School of Fujian Medical University, Fujian Cancer Hospital 66552 , Fuzhou, China; 4 College of Biological Science and Engineering, Fuzhou University, Fujian, China; 5 Department of Hepatobiliary and Pancreatic Surgery, Clinical Oncology School of Fujian Medical University, Fujian Cancer Hospital 66552 , Fuzhou, China; University of Manitoba, Winnipeg, Canada

**Keywords:** hepatocellular carcinoma, chronic hepatitis B virus, HBV-circRNA-5

## Abstract

Chronic hepatitis B virus (HBV) infection is regarded as one of the most serious infectious diseases and a significant global public health concern. Although the neonatal vaccine has been effective in impeding the transmission of HBV, tens of millions of HBV patients are still vulnerable to liver disease and even hepatocellular carcinoma (HCC). In this research, we demonstrated that HBV-encoded circRNA, designated as HBV-circRNA-5, was involved in the tumorigenesis of HCC. Overexpression of HBV-circRNA-5 promoted HCC cell proliferation and metastasis *in vitro* and promoted HCC tumor growth *in vivo*. Mechanistically, HBV-circRNA-5 bound to miR-9-3p selectively and resulted in the upregulation of YAP/TAZ expression, which consequently stimulated the proliferation of HCC. Overall, we provide new insight into understanding carcinogenesis and the progression of HBV-mediated HCC, as well as clarify a new cellular mechanism by which HBV promotes HCC progression through the HBV-circRNA-5/miR-9-3p/Hippo network.

**IMPORTANCE:**

HBV-mediated cirrhosis is considered a significant predisposing danger among HCC individuals despite the increasing incidence of HCC due to noninfectious factors. However, it is still unclear what molecular mechanisms underlie the emergence of HBV-mediated HCC. This study identifies HBV-encoded circular RNA HBV-circRNA-5 as a critical driver of HCC progression, with the capacity to promote HCC formation through the miR-9-3p/Hippo pathway. In summary, our study developed novel insights into understanding the molecular mechanism behind HBV-related HCC development and proposed a potential target for HCC treatment.

## INTRODUCTION

According to the WHO/IARC, HCC ranks as the fifth most prevalent malignant tumor while representing the second primary cause of carcinogenesis-related fatalities (1). HCC constitutes 80% of all liver cancer (2). Owing to the lack of early diagnostic markers and the subtlety of early symptoms associated with HCC, the majority of patients receive their diagnosis at a later stage, which hinders surgical intervention and results in a poor prognosis. Despite recent advances in targeted therapy, the long-term survival of HCC patients remained unsatisfactory (3).

HBV represents the foremost cause of cirrhosis and liver fibrosis, as well as HCC in China, contributing to roughly 190,000 HCC cases annually (4). HBV, an encapsulated virus, possesses an imperfect double-stranded circular DNA (rcDNA) structure (5). After endocytosis and fusion with cells, HBV releases nucleocapsid and migrates along microtubules to the nuclear periphery (6, 7). At the nuclear pore, the nucleocapsid disassembles, facilitating the transfer of rcDNA into the nucleus, where it undergoes conversion into covalently closed circular DNA necessary for viral RNA transcription (8). HBV has significant oncogenic capacity, which is associated with virus-induced epigenetic changes (9). The incorporation of the viral genome with the host genome results in the activation of the pro-carcinogenic gene (10). Furthermore, these host-virus fusion transcripts have been shown to affect HCC development through diverse mechanisms, such as inducing stem-cell-like characteristics, directly stimulating hepatocyte proliferation, and suppressing apoptosis (11, 12). In particular, the HBx encoded by HBV is another pivotal risk factor for HCC (13). It is well established that the function of HBx is linked to numerous gene and cell signaling pathway changes, such as ferroptosis, aerobic glycolysis, and autophagy (14–16).

A mounting volume of studies has demonstrated that noncoding RNA molecules played pivotal roles in the onset of HBV-related HCC (17–19). Long noncoding RNA (lncRNA) PCNAP1 was found to control HBV replication and accelerate HCC development (20). HOXA-AS2, which was regulated by HBV, promoted HCC proliferation and tumorigenesis by interacting with p53 and blocking the transcription of downstream genes (21). LINC01431 promoted PRMT1 accumulation on cccDNA and inhibited cccDNA transcription by regulating histone acetylation and H4R3me2a modifications (22).

Circular RNA (circRNA) is a recently identified noncoding RNA that rarely encodes proteins. The covalently closed-loop structure known as circRNA is produced by back-splicing pre-mRNA and lacks a 5′ end cap and a 3′ poly(A) tail (23, 24). CircRNAs are more stable in tissues or cells than typical linear RNAs, and RNA ribonuclease does not readily break them down (25, 26). Increasing evidence showed that circRNAs took part in diverse biological processes, including autophagy, oxidative stress, cell cycle dysregulation, and immunological responses (27–31). Association for circRNAs in cancer biology, including HCC, was also reported (32–34). A recent study proposed that HBV generated up to five distinct circRNAs based on computational prediction of viral circRNAs (35). However, it was still unclear whether these circRNAs were involved in HBV-related hepatocarcinogenesis. Previously, Zhu et al. (18) found that HBV_circ_1 increased the CDK1 expression and promoted the onset of HCC.

Initially reported in *Drosophila*, the Hippo signaling system was known to be crucial for organ development and homeostasis (36). The Hippo pathway is a complex biological system comprising multiple essential components, including YAP/TAZ, that collaborate to initiate a kinase cascade and activate transcriptional coactivators (37, 38). Dysregulation of YAP/TAZ is linked to several human diseases, including cancer (39, 40). For example, DUB1 promoted gastric cancer progression by deubiquitinating and stabilizing TAZ protein expression (41). SET1A promoted tumor development by increasing YAP methylation and nuclear localization (42). Notably, some circRNAs were found to be involved in regulating YAP/TAZ expression (43).

In this study, we characterized an HBV-encoded circRNA, named HBV-circRNA-5. We demonstrated that HBV-circRNA-5 promoted HCC proliferation and metastasis *in vitro*, as well as tumor formation in BALB/c nude mice. Importantly, in terms of mechanisms, we found that HBV-circRNA-5 could specifically bind to miR-9-3p and increased YAP/TAZ expression, which led to alterations in the YAP/TAZ-mediated Hippo signal pathway and HCC progression. Our findings not only improved the current understanding of the role of HBV-circRNA-5 in HBV-induced HCC progression but also provided potential therapeutic targets and prognostic indicators for the treatment of HCC.

## MATERIALS AND METHODS

### Patients and tissue samples

HCC tissue specimens utilized in this research were obtained from Fujian Provincial Cancer Hospital. HBV infection status was rigorously confirmed by using standard clinical diagnostic methods. Specifically, confirmation was primarily based on the detectable hepatitis B surface antigen (HbsAg >0.05 IU/mL) and HBV DNA viral loads (>10 IU/mL) in serum samples. HBV genotyping was not routinely performed as part of the standard clinical diagnostic protocol for these patients at the time of specimen collection. All of the samples used in this investigation were obtained with the patient’s informed consent and authorized under the Fujian Provincial Cancer Hospital’s Ethics Committee.

### Cell culture

We acquired both HepG2(RRID:CVCL_0027) and Hep3B(RRID:CVCL_0326) cells from Procell (Wuhan, China), and HepG2.2.15(RRID:CVCL_L855) was obtained from Meisen (Zhejiang, China). The SMMC-7721 cell line came from the Chinese Academy of Sciences (Shanghai, China). Hep3B was cultivated in EMEM medium, and the remaining cells in this study were maintained in DMEM medium with the incorporation of 10% FBS and were subsequently exposed to 37°C at an incubator.

### Total RNA extraction and real-time quantitative PCR

Total RNA from all samples was separated by TransZol reagent (Transgen, Beijing). After measuring RNA quality and concentration, 1 µg of RNA was generated into cDNA using the cDNA synthesis reagent (YEASEN, Shanghai). Using certain stem-loop primers, miRNA was generated into cDNA using miRNA reagent (Vazyme, Nanjing). Hieff qPCR Mix (YEASEN, Shanghai) and real-time PCR system (ThermoFisher) were then used to perform RT-qPCR. All gene data were normalized to the 18s rRNA and U6 values, and the target gene expression was computed using 2^−ΔΔCt^. All primers are listed in Table 1.

**TABLE 1:** The primers used in this study

Gene	Sequence (5′→3′）
HBV-circRNA-5-F	GGACTCATAAGCGTGGGACC
HBV-circRNA-5-R	TGCAATTTCCGTCCGAAGGT
HBV-F	CCTTCGGACGGAAATTGCAC
HBV-R	GCCCTACGAACCACTGAACA
18 s-F	AGAAACGGCTACCACATCC
18 s-R	CACCAGACTTGCCCTCCA
U6-F	CTCGCTTCGGCAGCACA
U6-R	AACGCTTCACGAATTTGCGT

### Cell transfection

Six-well plates were seeded with HCC cells and were cultured overnight. Subsequently, siRNA and expression plasmids for HBV-circRNA-5 were respectively delivered into cells by using TransIntro EL (Transgen, Beijing).

### Cell counting kit-8 proliferation assay

In 96-well plates, approximately 2,500 cells were seeded with 100 µL of complete media, and the plates were incubated for 24, 48, and 72 hours. Once CCK-8 buffer (MedChemExpress) was added to the plate, the orifice plate was incubated at 37°C. Lastly, the absorbance was estimated with the microplate reader, following the experimental well plates that had been processed for 1.5 hours.

### Cell invasion and migration assays

1 × 10^5^ cell precipitates were gently mixed containing 200 µL medium without any serum and were transferred into the top section of the transwell, while 800 µL complete media was placed into the bottom section. The transwell thereafter underwent incubation for 24–48 hours. According to migration assays, the invasion assay took place, with the exception that the transwell’s top section now contains Matrigel. After incubation for the indicated time, the top chamber was cleaned after the specified amount of time had passed, fixed with 4% cell fixative, and stained for 30 minutes with crystal violet.

### EdU (5-ethynyl-2'-deoxyuridine) assay

The EdU-594 kit (Beyotime, Shanghai) was used to perform the EdU test. In short, HCC cells were cultivated at 37°C after being seeded into the confocal dishes. Following the experimental intervention, cells were treated with fixative and treated with 10 µM EdU buffer for approximately 2 hours. After 30 minutes of Click Additive Solution incubation, the cells were stained with Hoechst for 10 minutes. Lastly, confocal laser scanning microscopy was used to take pictures.

### Colony formation assay

After seeding HCC cells in 6-well plates with 2 mL of complete medium, cells were kept for 15 to 20 days, with fresh medium replaced multiple times during the incubation period. Afterward, the medium was discarded, and the cells were washed twice with 1 × PBS. Cells were stained with crystal violet after being fixed with 4% cell fixative. ImageJ (RRID:SCR_003070) was used to count the cell colonies after they were captured on camera using a gel imaging technique.

### RNase R treatment

Three micrograms of RNA from HCC cells was treated with RNase R (GENESEED Guangzhou) and incubated for 8–10 minutes at 37°C. After that, RT-qPCR and RNA reverse transcription were carried out, as previously mentioned.

### FISH (fluorescence *in situ* hybridization) assay

Adhesive cells have been treated alongside fixative and then permeabilized by Triton X-100. The cells should be repeatedly washed with PBS before being incubated for 0.5 hours for pre-hybridization. The cells were then incubated with a hybridization containing the DIG-labeled probe (GENERAL BIOL, Anhui) targeting the junction site at 37℃ overnight. Cells were washed with SSC solutions at different concentrations and treated with anti-DIG-HRP at the ambient temperature. Subsequently, cells were treated for 20 minutes with DAPI and Cy3-tyramide. Finally, the cell’s fluorescence was examined with confocal laser scanning microscopy. The sequence of the DIG-labeled probe was 5′-TGCATGGTCCCACGCTTATGAGTCCAAGGAA-3′.

### Western blot

Following cell collection and two PBS washes, the pellets were broken down in lysis reagents (Beyotime, Shanghai) that contained a cocktail. Protein quantification was performed with Easy II Protein Quantitative Kit (BCA). After mixing protein samples with the 5× SDS PAGE Sample Loading Buffer, they were boiled for 10 minutes in a metal bath. After the proteins had been extracted on a separating protein gel, they were moved to a PVDF membrane that had been activated by 100% methanol. The membrane was blocked using a blocking solution. The membrane was washed with TBST and then treated with primary antibodies against TAZ (#83669, CST, RRID:AB_2800026), YAP (#14074, CST, RRID:AB_2650491), and β-actin (#4970, CST, RRID:AB_2223172) at 4℃ overnight with gentle shaking. Subsequently, a secondary antibody (HS101-01, transgene, RRID:AB_2629432) was added to the membrane and left to incubate for 1.5 hours at room temperature. Finally, three washes were performed on the membrane, the developer solution was treated, and a gel photography apparatus was used to photograph it.

### Animal experiment

All BALB/c nude mice (Spfbiotech, Beijing) were fed at least one week before the experiment. The HBV-circRNA-5 overexpression and control Hep3B pellets were collected and injected into nude mice with 1 × 10^7^ cells per mouse. The mice were sacrificed once the tumor volume reached 2,000 mm^3^. Then, the tumor size was measured and calculated by the formula: volume = 0.5 × length × width^2^. After the sacrifice of all mice, tumors were taken out, fixed with tissue fixative, and finally analyzed.

### Prediction of circRNA-miRNA interactions

VirusCircBase and RNAhybrid were used to predict miRNA binding with HBV-circRNA-5 in this study. In short, potential binding miRNAs were obtained from the VirusCircBase database. Next, we utilized RNAhybrid to predict the binding ability between HBV-circRNA-5 with each miRNA by calculating the minimum free energy (mfe threshold: −20 kcal/mol). Potential binding sites between HBV-circRNA-5 and miRNA were also provided by RNAhybrid.

### Dual-luciferase reporter assays

The full length of HBV-circRNA-5 (circRNA5 WT) and its mutation (circRNA5 MUT) were inserted into the pmirGLO Vector (PROMEGA, RRID: Addgene_212613). Then, all plasmids were co-transfected into HEK-293T with miR-9-3p. After collecting cell pellets, they were lysed in a 1× cell lysis buffer. Afterward, the supernatant was centrifuged, and the sample reading was assessed using the Luciferase Kit (YEASEN, Shanghai).

### Statistical analysis

GraphPad Prism 6 was used to perform statistical analysis for this study. Mean ± SD was utilized to display the data. Statistical significance between the two groups was assessed using Student’s *t*-tests, and the differences between multiple groups were examined using two-way ANOVA. *P*-values < 0.05 were considered to be significant (*P < 0.05, **P < 0.01, and ***P < 0.001).

## RESULTS

### Characterization of HBV-circRNA-5 in HCC

**Fig 1:**
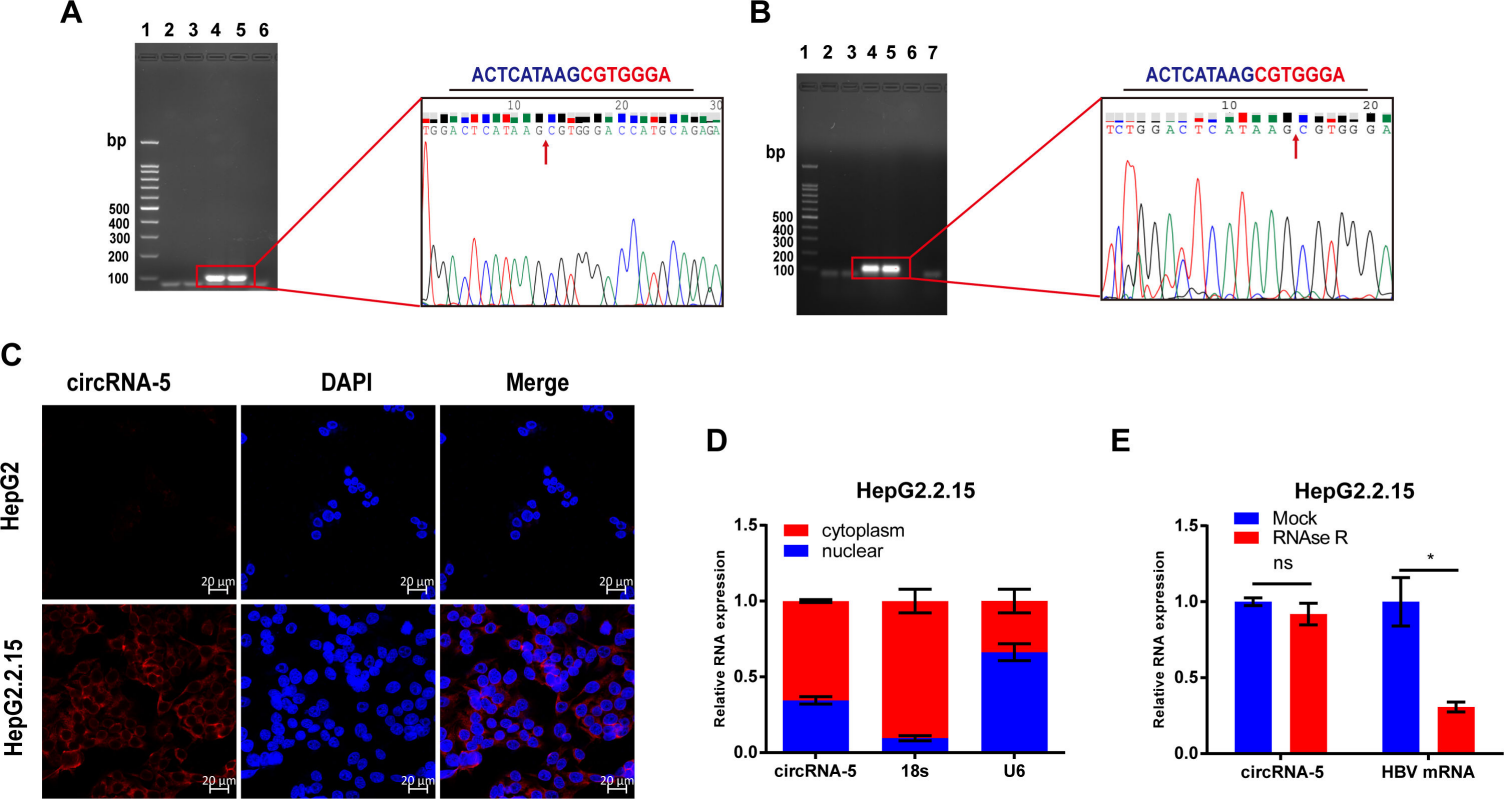
Characterization of HBV-circRNA-5 in HCC tissue and cell. (**A and B**) Presence of HBV-circRNA-5 in HBV-positive HCC tissues (A, Lan1: DNA ladder, Lan2-3:HBV-negative tissue, Lna4-5: HBV-positive tissue, Lan6:H_2_O) and HepG2.2.15 (B, Lan1: DNA ladder, Lan2-3:HepG2, Lna4-5: HepG2.2.15, Lan7:H_2_O) was validated by Sanger sequencing. (**C and D**) HBV-circRNA-5 localization in HepG2.2.15 was detected by FISH (**C**) and subcellular fractionation assay (**D**). (**E**) Stability for HBV-circRNA-5 and HBV RNA was evaluated through RNase R treatment assay. n=3 biologically independent samples. Data were quantified as mean ± SD. (ns indicated no significance, *P < 0.05).

HBV-circRNA-5 was found in the HBV genome’s nucleotide sequence between 505 and 2,471 (35). To further examine the circularization of HBV-circRNA-5, splice junction overlapping divergent primers (Sjod Primers) targeting the spliced junction were designed to amplify the circularized site. According to the RT-qPCR results, HBV-circRNA-5 was amplified from HBV-related HCC tissue in comparison to negative control tissue cDNA (Fig. 1A, left). Further results from Sanger sequencing of the qPCR results confirmed the expected cyclization site sequences (Fig. 1A, right). The same results were also found in the HepG2.2.15 cell line (Fig. 1B). FISH and subcellular localization assays confirmed that HBV-circRNA-5 was mostly located in the cytoplasm (Fig. 1C and D). Moreover, we also examined the stability of HBV-circRNA-5 formation by RNase R. Our data showed that HBV-circRNA-5 was much more resistant to degradation than HBV mRNA (Fig. 1E). Taken together, we demonstrated the presence of virus-encoded HBV-circRNA-5 in HBV-related HCC.

### Knockdown of HBV-circRNA-5 inhibited malignancy in HBV-related HCC

**Fig 2:**
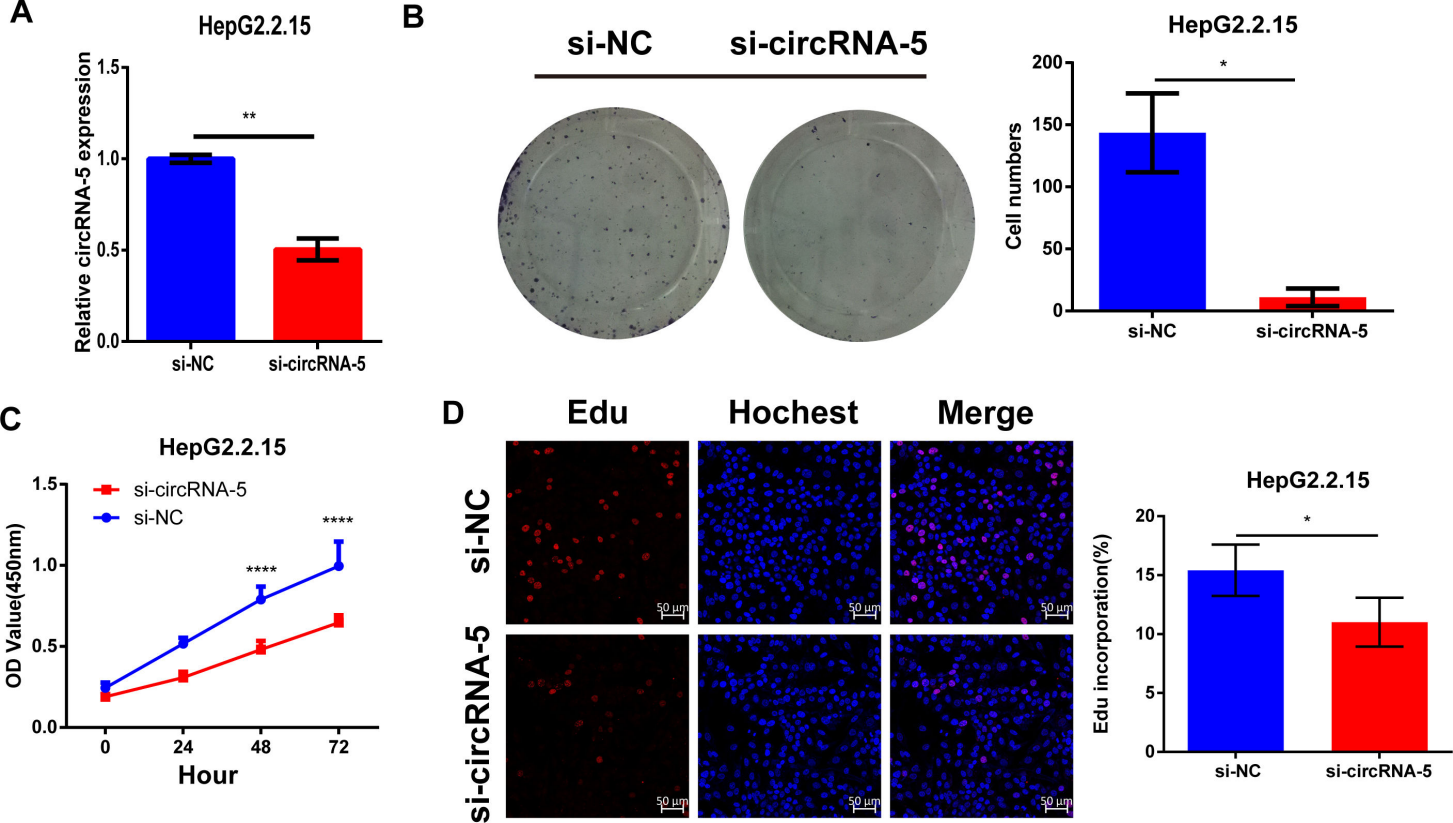
HBV-circRNA-5 knockdown inhibited HCC proliferation. (**A**) The impact of siRNA was assessed through RT-qPCR. n=3 biologically independent samples. Colony formation (**B**), CCK8 (**C**), and EdU (**D**) were implemented to evaluate the potential role of HBV-circRNA-5 in HCC proliferation through siRNA-induced knockdown. n=3 biologically independent samples. Data were quantified as mean ± SD. (*P < 0.05, **P < 0.01, and ***P < 0.001).

To test its involvement in oncogenesis in HBV-related HCC, we silenced HBV-circRNA-5 expression using siRNA targeting the junction site. As seen in Fig. 2A, the expression of HBV-circRNA-5 significantly decreases according to siRNA treatment. Subsequently, we found that HBV-circRNA-5 knockdown inhibited HepG2.2.15 proliferation, as tested by colony formation and CCK-8 assay (Fig. 2B and C). The EdU test showed that cell growth significantly decreased due to HBV-circRNA-5 knockdown (Fig. 2D). Together, these data indicated that HBV-circRNA-5 with low expression inhibited malignant biological behaviors in HBV-positive HCC cells.

### HBV-circRNA-5 promoted HCC proliferation, migration, and invasion

**Fig 3:**
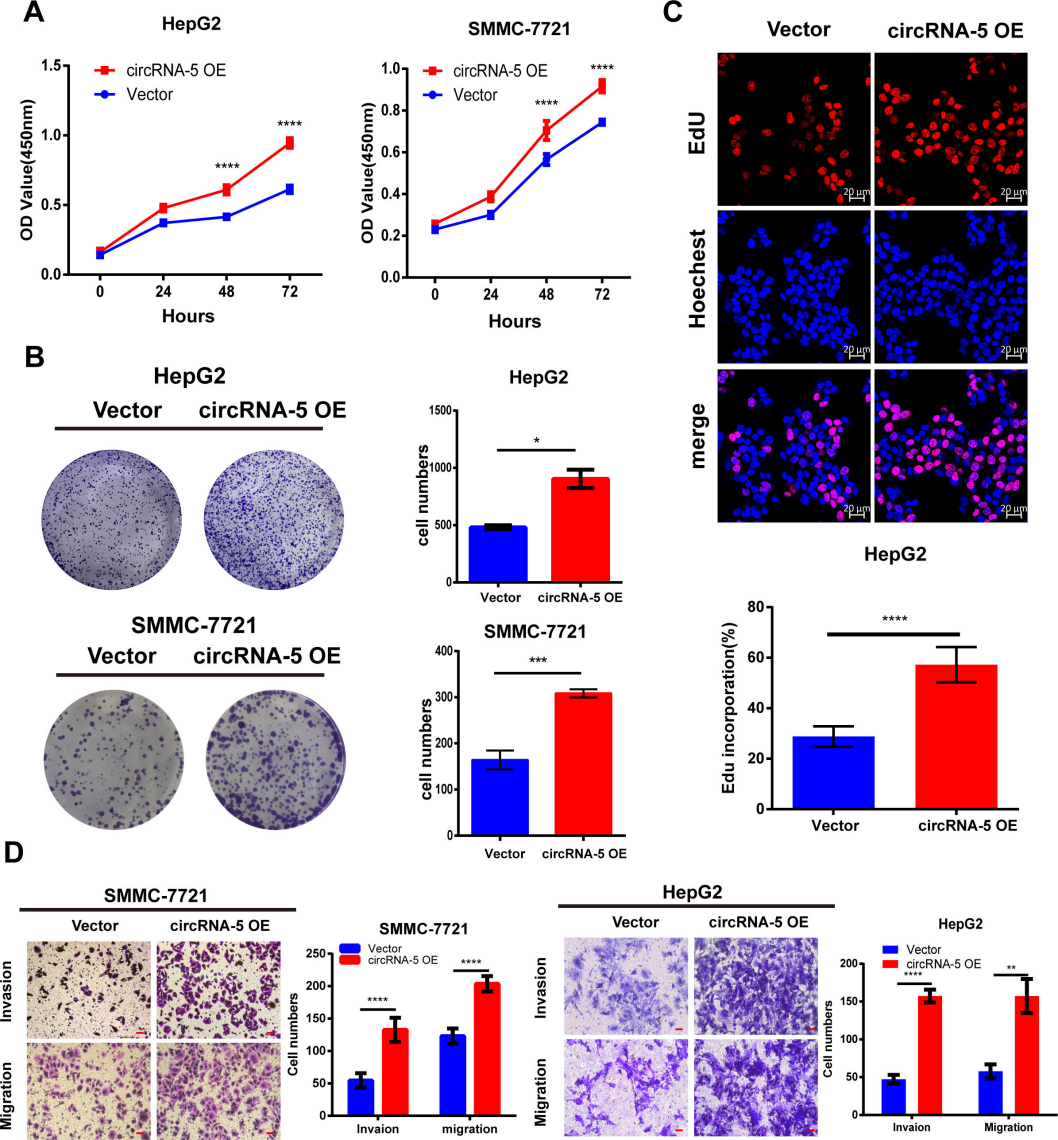
HBV-circRNA-5 OE promoted HCC proliferation, invasion, and migration. CCK8 (**A**), colony formation (**B**), and EdU (**C**) were implemented for evaluating the HCC cell proliferation after HBV-circRNA-5 OE. n=3 biologically independent samples. (**D**) Transwell assay was implemented to evaluate cell invasion and migration induced by HBV-circRNA-5 OE. Scale bar = 40 µm. n=3 biologically independent samples. Data were quantified as mean ± SD. (*P < 0.05, **P < 0.01, and ***P < 0.001).

In parallel, we investigated the capacity for HBV-circRNA-5 overexpression (OE) on HCC cells. The full-length sequence of HBV-circRNA-5 was constructed into an expression vector and transfected into cells. The proliferative capacity of highly expressed HBV-circRNA-5 on HCC cells was detected through CCK-8 assays. The results revealed that HBV-circRNA-5 significantly boosted HCC proliferation (Fig. 3A). The results of plate cloning assays demonstrated that HBV-circRNA-5 significantly augmented the capacity of HCC to generate clones (Fig. 3B). Furthermore, EdU assays showed that HBV-circRNA-5 increased the number of EdU-positive cells (Fig. 3C). Finally, we investigated whether HBV-circRNA-5 affected the HCC invasion and migration. As expected, HBV-circRNA-5 OE significantly promoted the metastasis of HCC *in vitro* (Fig. 3D). In general, HBV-circRNA-5 OE played a remarkable role in the formation and spread of HCC by encouraging tumor growth.

### HBV-circRNA-5 acted as the sponge for miR-9-3p

**Fig 4:**
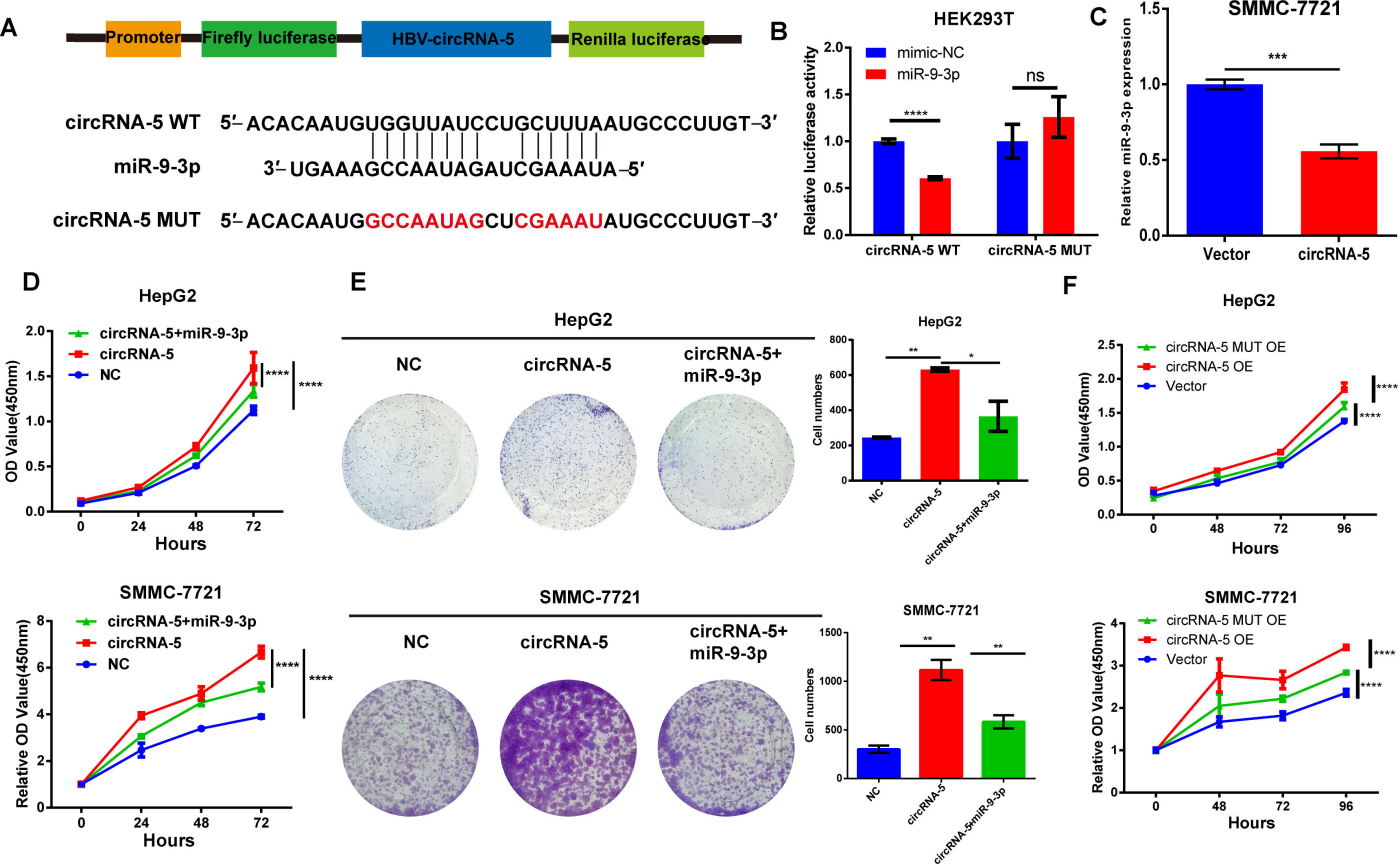
HBV-circRNA-5 served as miR-9-3p sponge. (**A**) Schematic illustration of HBV-circRNA-5 luciferase reporter vector construction. (**B**) Luciferase assays were implemented in evaluating the interaction between HBV-circRNA-5 and miR-9-3p. n=3 biologically independent samples. (**C**) miR-9-3p expression was evaluated in SMMC-7721 after transfection with HBV-circRNA-5. CCK-8 (**D**) and colony formation (**E**) were implemented in evaluating the HCC proliferation after transfection with miR-9-3p mimics, empty vector, HBV-circRNA-5 vector, and mimics NC. n=3 biologically independent samples. (**F**) CCK-8 was implemented in evaluating the HCC proliferation after HBV-circRNA-5 OE and MUT OE. Data were quantified as mean ± SD. (*P < 0.05, **P < 0.01, and ***P < 0.001).

To elucidate the cellular mechanism for HBV-circRNA-5 to control the development of HCC, we explored the miRNAs that interacted with HBV-circRNA-5. According to VirusCircBase data, HBV-circRNA-5 was considered to bind with multiple distinct miRNAs, including miR-9-3p, which has been shown to play a crucial role in HCC development (35, 44). RNAhybrid was used to predict the binding sites (Fig. 4A). To verify circRNA-5/miR-9-3p binding, we inserted full-length HBV-circRNA-5 (WT and MUT) into the reporter gene plasmid and co-transfected it into HEK-293T cells together with miR-9-3p. Results showed that the luciferase activity in the HBV-circRNA-5 WT co-transfected group was comparatively lower than the control. However, there was no significant difference in the HBV-circRNA-5 MUT co-transfected group than the control, which confirmed HBV-circRNA-5 and miR-9-3p integration (Fig. 4B). Additionally, we found that HBV-circRNA-5 OE dramatically reduced miR-9-3p expression, indicating HBV-circRNA-5 as a miR-9-3p sponge (Fig. 4C). Then, we checked whether HBV-circRNA-5 regulated HCC progression through the miR-9-3p sponge mechanism. The results of CCK-8 and plate cloning assays demonstrated that the promotion of HCC proliferation by HBV-circRNA-5 could be reversed by miR-9-3p mimics (Fig. 4D and E). To interrogate the important role of circRNA-5/miR-9-3p binding, we engineered a circRNA-5 mutant with mismatches to disrupt its binding to miR-9-3p. Next, we performed a proliferation assay by overexpressing circRNA5-MUT alongside circRNA5-WT. The results are shown in Fig. 4F. Compared to circRNA5-WT, the ability for circRNA5-MUT to promote HCC cell proliferation is noticeably reduced. However, circRNA5-MUT promoted HCC proliferation as well, which indicated that circRNA-5 might engage additional molecular targets beyond miR-9-3p to regulate cell growth.

### HBV-circRNA-5 promoted HCC progression by regulating the Hippo signaling pathway through miR-9-3p

**Fig 5:**
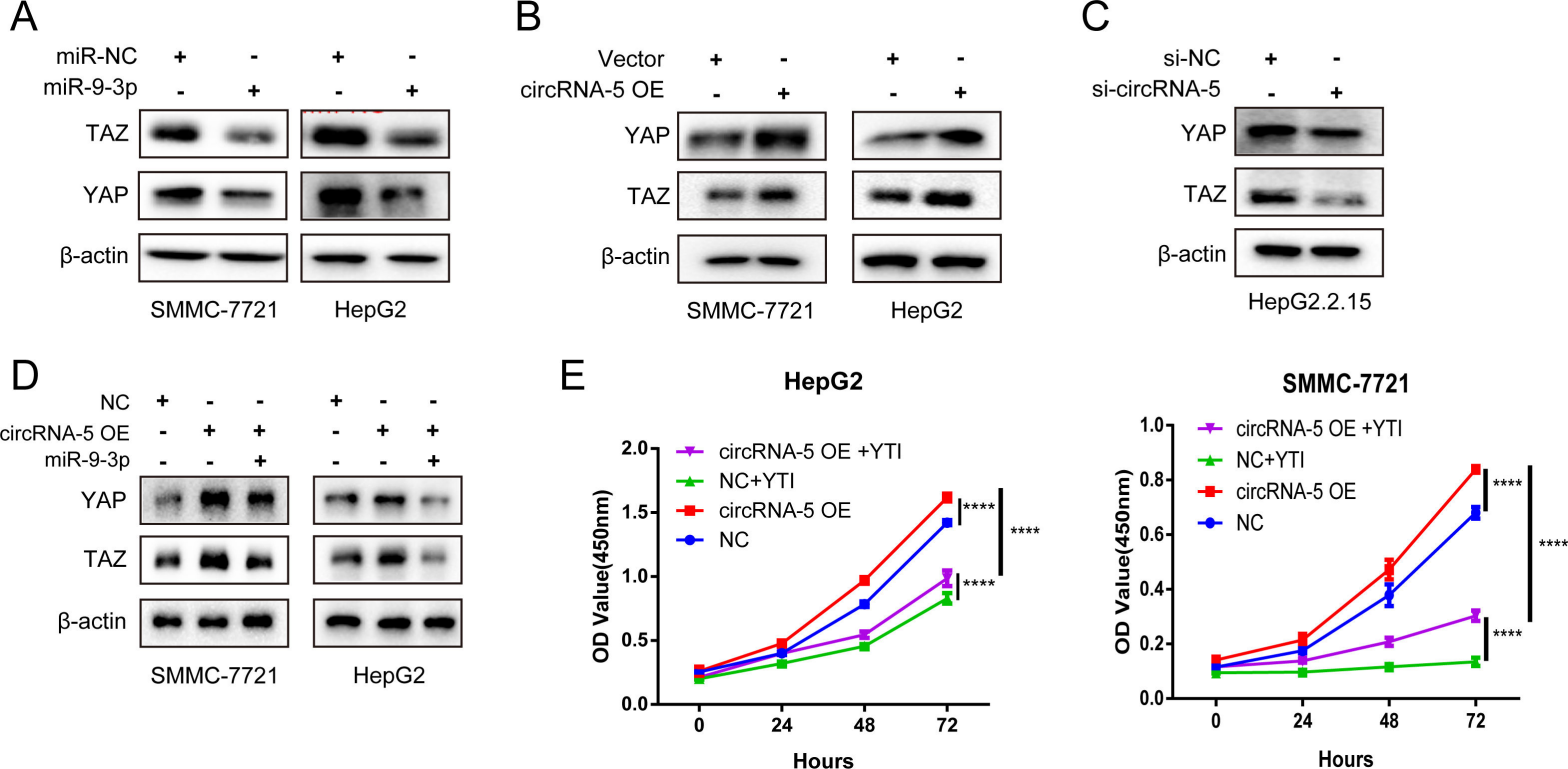
HBV-circRNA-5 promoted HCC progression by regulating the Hippo pathway via targeting miR-9-3p. (**A**) YAP/TAZ protein levels in HCC cells transfected with miR-9-3p mimics and NC mimics. (**B and C**) The protein levels of YAP/TAZ were observed in HCC cells by HBV-circRNA-5 OE (**B**) and knockdown (**C**). (**D**) YAP/TAZ protein levels in HCC transfected with either miR-9-3p, HBV-circRNA-5 vector, and NC. (**E**) CCK-8 assay was implemented in evaluating the HCC proliferation transfected with an empty vector and HBV-circRNA-5 vector after the addition of YTI. n=3 biologically independent samples. Data were quantified as mean ± SD. (*P < 0.05, **P < 0.01, and ***P < 0.001).

We are committed to investigating the possible role of the HBV-circRNA-5/miR-9-3p axis in HCC oncogenesis. It has been certified that miR-9-3p played a tumor-preventive role in HCC by regulating the Hippo pathway (44). We first checked the effectiveness of miR-9-3p affecting YAP/TAZ expression. The results revealed that miR-9-3p dramatically reduced the production of the proteins YAP and TAZ (Fig. 5A). As expected, upregulation of YAP/TAZ protein expression was observed following HBV-circRNA-5 OE in HCC cells (Fig. 5B). By contrast, knocking down HBV-circRNA-5 expression in HepG2.2.15 cells attenuated YAP/TAZ protein expression (Fig. 5C). Importantly, the promotional effects of YAP/TAZ expression induced by HBV-circRNA-5 OE were reversed by miR-9-3p mimics (Fig. 5D). Finally, we treated SMMC-7721 and HepG2 cells with YAP/TAZ inhibitor-1 (YTI) together with HBV-circRNA-5 OE. The CCK-8 results showed that YTI effectively attenuated HBV-circRNA-5 promotion for HCC proliferation (Fig. 5E). Collectively, these findings indicated that HBV-circRNA-5 promotes HCC proliferation through the HBV-circRNA-5/miR-9-3p/Hippo signaling pathway.

### HBV-circRNA-5 promotes tumor growth *in vivo*

**Fig 6:**
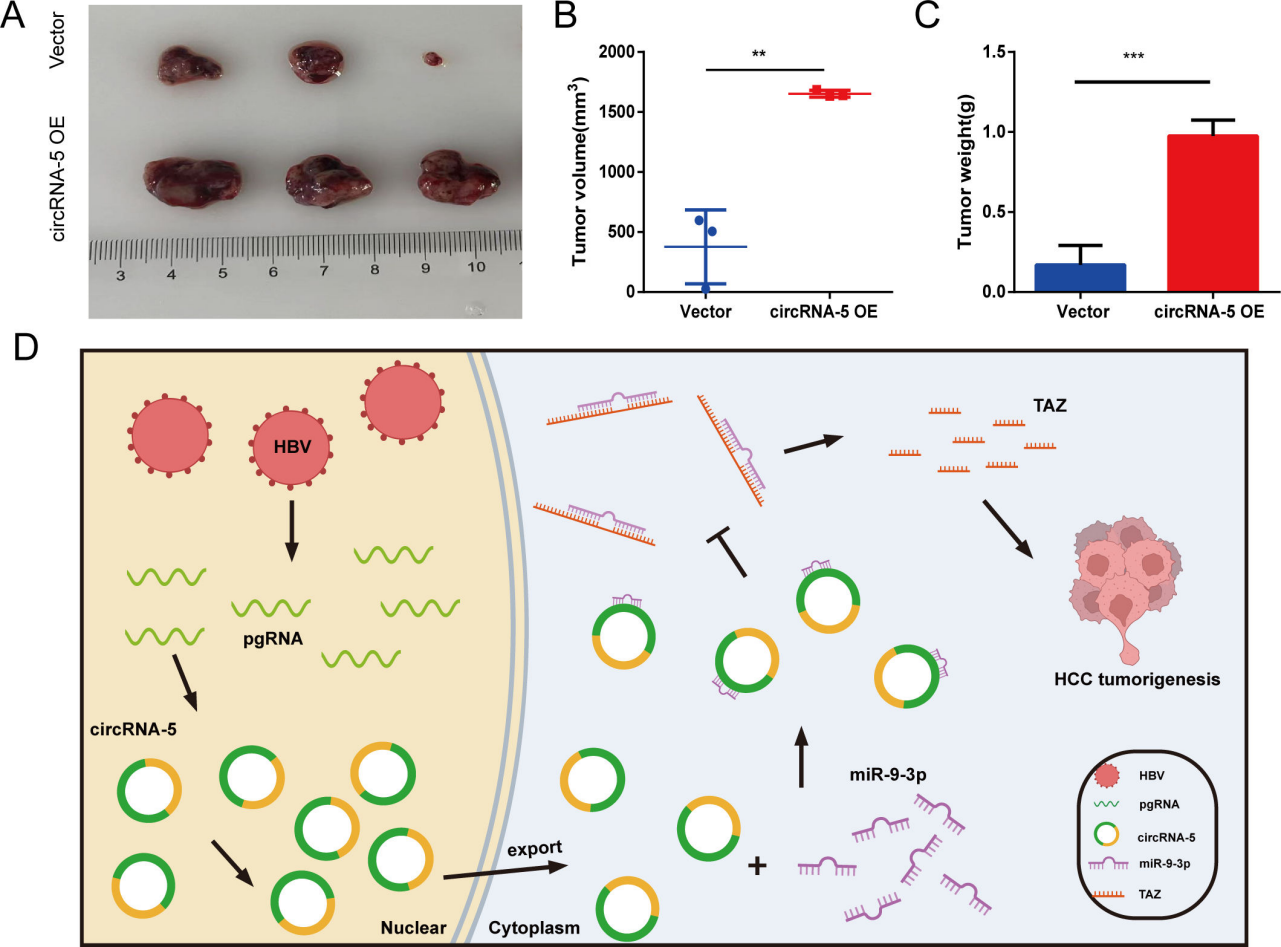
HBV-circRNA-5 promoted tumor growth *in vivo*. (**A**) Subcutaneous tumors in vector as well as HBV-circRNA-5 OE groups. 3 mice/group. (**B and C**) Tumor volume (**B**) and weight (**C**) from vector and HBV-circRNA-5 OE groups were measured after removing the tumor. n=3 biologically independent samples. (**D**) A novel molecular mechanism behind the development of HBV-associated HCC. Data were quantified as mean ± SD. (*P < 0.05, **P < 0.01, and ***P < 0.001).

To verify the modulated HBV-circRNA-5 upon the *in vivo* development and formation of HCC, a series of experiments were conducted. Initially, Hep3B cells with stable expression of HBV-circRNA-5 were constructed, after which the precipitated cells were injected into nude mice (Fig. 6A). Findings demonstrated that HBV-circRNA-5 OE produced substantially more subcutaneous tumor volume and weight than the control (Fig. 6B and C).

## DISCUSSION

Up to now, HBV-mediated cirrhosis is considered a significantly predisposing danger among HCC individuals despite the increasing incidence of HCC due to noninfectious factors (45, 46). It has been shown that HBV can cause HCC directly or indirectly (47, 48). In the case of indirect regulation, HBV primarily promotes hepatocarcinogenesis by encoding a protein known as HBx (49). Although cellular mechanisms of HBV-mediated HCC remain incompletely understood, the advancement of high-throughput sequencing technology has opened up new research possibilities in this area. Several investigations have indicated that viral HBV could aid in the HCC advancement through encoding and generating novel molecules. For instance, Zhao et al. (50) identified that HBV-miR-3 regulated HBV self-replication. Moreover, HBV-miR-3 promotes HCC growth by hybridizing with PPM1A mRNA (51). In this study, we revealed the involvement of HBV-encoded circRNA-5 in HCC oncogenesis. HBV-circRNA-5 was more stable than linear mRNA and predominantly localized in the cytoplasm. Functional *in vitro* studies showed that HBV-circRNA-5 promoted HCC proliferation, invasion, and migration. Similarly, HBV-circRNA-5 stimulated tumor expansion *in vivo*. Given the evidence, we proposed HBV-circRNA-5 as a cancer-promoting molecule, providing a potential therapeutic target for HCC treatment.

The competing endogenous RNA (ceRNA) network, a key post-transcriptional regulatory mechanism in cells, has been widely linked to numerous biological processes. CircRNAs are a crucial part of the ceRNA regulatory network. They can bind to miRNAs and are extensively involved in cancer development by regulating the circRNA-miRNA-mRNA signaling axis. CircRPN2 functions to be the competitive circRNA targeting miR-183-5p, inhibiting glucose metabolism and tumor development by increasing FOXO1 expression (52). Our study identified miR-9-3p as a potential target of HBV-circRNA-5. Dual fluorescein reporter assay and RT-qPCR confirmed that HBV-circRNA-5 binds with miR-9-3p and reduces its expression. The stimulation of HBV-circRNA-5 on HCC proliferation was successfully mitigated by miR-9-3p. Previous research demonstrated that miR-9-3p might be vital in controlling the Hippo pathway. We proved here that HBV-circRNA-5 increased the YAP/TAZ expression. Additionally, we discovered that YAP/TAZ inhibitors prevented HBV-circRNA-5 from promoting HCC proliferation. According to these findings, we declared that HBV-circRNA-5 performed the role of miR-9-3p sponges and took part in the HCC onset by interfering with the Hippo pathway.

Although our study demonstrated the putative role of HBV-circRNA-5 in facilitating HCC progression by regulating the Hippo signaling pathway, it is essential to consider several potential limitations when interpreting our findings. A noteworthy question is whether the regulatory mechanism of HBV-circRNA-5 applies to other HBV genotypes besides genotype D. HBV genotypes play important roles in HBV pathogenesis, while presenting different distribution characteristics. HBV genotype C is more likely to lead to liver cirrhosis and ultimately HCC (53, 54). Novel recombinant HBV between genotypes B and C exhibits greater virulence and is associated with a poorer prognosis in HCC patients (55). HBV-circRNA-5 was initially identified as originating from genotype D (35). The HBV-producing cell line, HepG2.2.15, which was utilized to estimate the involvement of HBV-circRNA-5 in oncogenesis in HCC, was also derived from HBV genotype D. Subsequently, all the constructs utilized in our study were designed according to HBV genotype D. Whether our findings are consistent across all subtypes may need to be tested. Another important consideration is the potential for off-target or nonspecific effects of the HBV-circRNA-5 regulatory mode in HCC. Our findings established a crucial functional axis involving HBV-circRNA-5 sponging of miR-9-3p to regulate the Hippo signaling axis in HBV-related HCC. However, it is important to acknowledge the inherent complexity of circRNA biology. Similar to many characterized circRNAs acting as miRNA sponges, HBV-circRNA-5 likely possesses multiple miRNA binding sites, enabling it to potentially sequester a broader repertoire of miRNAs beyond miR-9-3p. While our rescue experiments strongly implicated the HBV-circRNA-5/miR-9-3p/Hippo signaling axis as a major contributor to the observed phenotypes, circRNA5-MUT retained the ability to promote HCC proliferation. Therefore, the results indicated that circRNA-5 might engage off-target or nonspecific effects on HCC proliferation, involve contributions from other interacting miRNAs, or indirectly affect pathways, all of which warrant further investigation.

In conclusion, we confirmed the presence of HBV-circRNA-5 and established the oncogenic function in HBV-related HCC. We clarified that HBV-circRNA-5 stimulated HCC formation via the miR-9-3p/Hippo signaling axis. Our study developed novel insights into understanding the molecular mechanism behind HBV-related HCC development and provided a new framework for elucidating the carcinogenic mechanisms of multigenotypic HBV infections.

## Data Availability

Data from the present study are available upon request.

## References

[1] Sung H, Ferlay J, Siegel RL, Laversanne M, Soerjomataram I, Jemal A, Bray F. 2021. Global cancer statistics 2020: GLOBOCAN estimates of incidence and mortality worldwide for 36 Cancers in 185 Countries. CA Cancer J Clin 71:209–249. doi:10.3322/caac.21660 33538338

[2] Chrysavgis L, Giannakodimos I, Diamantopoulou P, Cholongitas E. 2022. Non-alcoholic fatty liver disease and hepatocellular carcinoma: clinical challenges of an intriguing link. World J Gastroenterol 28:310–331. doi:10.3748/wjg.v28.i3.310 35110952 PMC8771615

[3] Kudo M, Finn RS, Qin S, Han KH, Ikeda K, Piscaglia F, Baron A, Park JW, Han G, Jassem J, Blanc JF, Vogel A, Komov D, Evans TRJ, Lopez C, Dutcus C, Guo M, Saito K, Kraljevic S, Tamai T, Ren M, Cheng AL. 2018. Lenvatinib versus sorafenib in first-line treatment of patients with unresectable hepatocellular carcinoma: a randomised phase 3 non-inferiority trial. Lancet 391:1163–1173. doi:10.1016/S0140-6736(18)30207-1 29433850

[4] Diseases GBD, Injuries C. 2020. Global burden of 369 diseases and injuries in 204 countries and territories, 1990-2019: a systematic analysis for the Global Burden of Disease Study 2019. Lancet 396:1204–1222. doi:10.1016/S0140-6736(20)30925-9 33069326 PMC7567026

[5] Tong S, Revill P. 2016. Overview of hepatitis B viral replication and genetic variability. J Hepatol 64:S4–S16. doi:10.1016/j.jhep.2016.01.027 27084035 PMC4834849

[6] Macovei A, Petrareanu C, Lazar C, Florian P, Branza-Nichita N. 2013. Regulation of hepatitis B virus infection by Rab5, Rab7, and the endolysosomal compartment. J Virol 87:6415–6427. doi:10.1128/JVI.00393-13 23536683 PMC3648082

[7] Rabe B, Glebe D, Kann M. 2006. Lipid-mediated introduction of hepatitis B virus capsids into nonsusceptible cells allows highly efficient replication and facilitates the study of early infection events. J Virol 80:5465–5473. doi:10.1128/JVI.02303-05 16699026 PMC1472160

[8] Xia Y, Guo H. 2020. Hepatitis B virus cccDNA: Formation, regulation and therapeutic potential. Antiviral Res 180:104824. doi:10.1016/j.antiviral.2020.104824 32450266 PMC7387223

[9] Jiang Y, Han Q, Zhao H, Zhang J. 2021. The mechanisms of HBV-induced hepatocellular carcinoma. J Hepatocell Carcinoma 8:435–450. doi:10.2147/JHC.S307962 34046368 PMC8147889

[10] Levrero M, Zucman-Rossi J. 2016. Mechanisms of HBV-induced hepatocellular carcinoma. J Hepatol 64:S84–S101. doi:10.1016/j.jhep.2016.02.021 27084040

[11] Jiang S, Yang Z, Li W, Li X, Wang Y, Zhang J, Xu C, Chen PJ, Hou J, McCrae MA, Chen X, Zhuang H, Lu F. 2012. Re-evaluation of the carcinogenic significance of hepatitis B virus integration in hepatocarcinogenesis. PLoS One 7:e40363. doi:10.1371/journal.pone.0040363 22962577 PMC3433482

[12] An P, Xu J, Yu Y, Winkler CA. 2018. Host and viral genetic variation in HBV-related hepatocellular carcinoma. Front Genet 9:261. doi:10.3389/fgene.2018.00261 30073017 PMC6060371

[13] Dong ML, Wen X, He X, Ren JH, Yu HB, Qin YP, Yang Z, Yang ML, Zhou CY, Zhang H, Cheng ST, Chen J. 2022. HBx mediated increase of DDX17 contributes to HBV-related hepatocellular carcinoma tumorigenesis. Front Immunol 13:871558. doi:10.3389/fimmu.2022.871558 35784274 PMC9243429

[14] Lei Y, Xu X, Liu H, Chen L, Zhou H, Jiang J, Yang Y, Wu B. 2021. HBx induces hepatocellular carcinogenesis through ARRB1-mediated autophagy to drive the G_1_/S cycle. Autophagy 17:4423–4441. doi:10.1080/15548627.2021.1917948 33866937 PMC8726737

[15] Liu GZ, Xu XW, Tao SH, Gao MJ, Hou ZH. 2021. HBx facilitates ferroptosis in acute liver failure via EZH2 mediated SLC7A11 suppression. J Biomed Sci 28:67. doi:10.1186/s12929-021-00762-2 34615538 PMC8495979

[16] Chen L, Lin X, Lei Y, Xu X, Zhou Q, Chen Y, Liu H, Jiang J, Yang Y, Zheng F, Wu B. 2022. Aerobic glycolysis enhances HBx-initiated hepatocellular carcinogenesis via NF-κBp65/HK2 signalling. J Exp Clin Cancer Res 41:329. doi:10.1186/s13046-022-02531-x 36411480 PMC9677649

[17] Sartorius K, An P, Winkler C, Chuturgoon A, Li X, Makarova J, Kramvis A. 2021. The epigenetic modulation of cancer and immune pathways in hepatitis B Virus-associated hepatocellular carcinoma: the influence of HBx and miRNA dysregulation. Front Immunol 12:661204. doi:10.3389/fimmu.2021.661204 33995383 PMC8117219

[18] Zhu M, Liang Z, Pan J, Zhang X, Xue R, Cao G, Hu X, Gong C. 2021. Hepatocellular carcinoma progression mediated by hepatitis B virus-encoded circRNA HBV_circ_1 through interaction with CDK1. Mol Ther Nucleic Acids 25:668–682. doi:10.1016/j.omtn.2021.08.011 34589285 PMC8463320

[19] Sarfaraz N, Somarowthu S, Bouchard MJ. 2023. The interplay of long noncoding RNAs and hepatitis B virus. J Med Virol 95:e28058. doi:10.1002/jmv.28058 35946066

[20] Feng J, Yang G, Liu Y, Gao Y, Zhao M, Bu Y, Yuan H, Yuan Y, Yun H, Sun M, Gao H, Zhang S, Liu Z, Yin M, Song X, Miao Z, Lin Z, Zhang X. 2019. LncRNA PCNAP1 modulates hepatitis B virus replication and enhances tumor growth of liver cancer. Theranostics 9:5227–5245. doi:10.7150/thno.34273 31410212 PMC6691589

[21] Liu N, Liu Q, Yang X, Zhang F, Li X, Ma Y, Guan F, Zhao X, Li Z, Zhang L, Ye X. 2018. Hepatitis B virus-upregulated LNC-HUR1 promotes cell proliferation and tumorigenesis by blocking p53 activity. Hepatology 68:2130–2144. doi:10.1002/hep.30098 29790592

[22] Sun Y, Teng Y, Wang L, Zhang Z, Chen C, Wang Y, Zhang X, Xiang P, Song X, Lu J, Li N, Gao L, Liang X, Xia Y, Wu Z, Ma C. 2022. LINC01431 promotes histone H4R3 methylation to impede HBV covalently closed circular DNA transcription by stabilizing PRMT1. Adv Sci (Weinh) 9:e2103135. doi:10.1002/advs.202103135 35398991 PMC9165498

[23] Jeck WR, Sorrentino JA, Wang K, Slevin MK, Burd CE, Liu J, Marzluff WF, Sharpless NE. 2013. Circular RNAs are abundant, conserved, and associated with ALU repeats. RNA 19:141–157. doi:10.1261/rna.035667.112 23249747 PMC3543092

[24] Santer L, Bär C, Thum T. 2019. Circular RNAs: a novel class of functional RNA molecules with a therapeutic perspective. Mol Ther 27:1350–1363. doi:10.1016/j.ymthe.2019.07.001 31324392 PMC6697450

[25] Li D, Li Z, Yang Y, Zeng X, Li Y, Du X, Zhu X. 2020. Circular RNAs as biomarkers and therapeutic targets in environmental chemical exposure-related diseases. Environ Res 180:108825. doi:10.1016/j.envres.2019.108825 31683121

[26] Li Y, Ge Y, Xu L, Jia R. 2020. Circular RNA ITCH: a novel tumor suppressor in multiple cancers. Life Sci (1962) 254:117176. doi:10.1016/j.lfs.2019.117176 31843532

[27] Dai X, Chen C, Yang Q, Xue J, Chen X, Sun B, Luo F, Liu X, Xiao T, Xu H, Sun Q, Zhang A, Liu Q. 2018. Exosomal circRNA_100284 from arsenite-transformed cells, via microRNA-217 regulation of EZH2, is involved in the malignant transformation of human hepatic cells by accelerating the cell cycle and promoting cell proliferation. Cell Death Dis 9:454. doi:10.1038/s41419-018-0485-1 29674685 PMC5908808

[28] Zhao Q, Liu J, Deng H, Ma R, Liao JY, Liang H, Hu J, Li J, Guo Z, Cai J, Xu X, Gao Z, Su S. 2020. Targeting mitochondria-located circRNA SCAR alleviates NASH via reducing mROS output. Cell 183:76–93. doi:10.1016/j.cell.2020.08.009 32931733

[29] Wang Y, Mo Y, Peng M, Zhang S, Gong Z, Yan Q, Tang Y, He Y, Liao Q, Li X, Wu X, Xiang B, Zhou M, Li Y, Li G, Li X, Zeng Z, Guo C, Xiong W. 2022. The influence of circular RNAs on autophagy and disease progression. Autophagy 18:240–253. doi:10.1080/15548627.2021.1917131 33904341 PMC8942425

[30] Zheng H, Huang S, Wei G, Sun Y, Li C, Si X, Chen Y, Tang Z, Li X, Chen Y, Liao W, Liao Y, Bin J. 2022. CircRNA Samd4 induces cardiac repair after myocardial infarction by blocking mitochondria-derived ROS output. Mol Ther 30:3477–3498. doi:10.1016/j.ymthe.2022.06.016 35791879 PMC9637749

[31] Amaya L, Grigoryan L, Li Z, Lee A, Wender PA, Pulendran B, Chang HY. 2023. Circular RNA vaccine induces potent T cell responses. Proc Natl Acad Sci USA 120:e2302191120. doi:10.1073/pnas.2302191120 37155869 PMC10193964

[32] Huang G, Liang M, Liu H, Huang J, Li P, Wang C, Zhang Y, Lin Y, Jiang X. 2020. CircRNA hsa_circRNA_104348 promotes hepatocellular carcinoma progression through modulating miR-187-3p/RTKN2 axis and activating Wnt/β-catenin pathway. Cell Death Dis 11:1065. doi:10.1038/s41419-020-03276-1 33311442 PMC7734058

[33] Xu J, Ji L, Liang Y, Wan Z, Zheng W, Song X, Gorshkov K, Sun Q, Lin H, Zheng X, Chen J, Jin R, Liang X, Cai X. 2020. CircRNA-SORE mediates sorafenib resistance in hepatocellular carcinoma by stabilizing YBX1. Sig Transduct Target Ther 5:298. doi:10.1038/s41392-020-00375-5 PMC7762756 33361760

[34] Song R, Ma S, Xu J, Ren X, Guo P, Liu H, Li P, Yin F, Liu M, Wang Q, Yu L, Liu J, Duan B, Rahman NA, Wołczyński S, Li G, Li X. 2023. A novel polypeptide encoded by the circular RNA ZKSCAN1 suppresses HCC via degradation of mTOR. Mol Cancer 22:16. doi:10.1186/s12943-023-01719-9 36691031 PMC9869513

[35] Cai Z, Fan Y, Zhang Z, Lu C, Zhu Z, Jiang T, Shan T, Peng Y. 2021. VirusCircBase: a database of virus circular RNAs. Brief Bioinform 22:2182–2190. doi:10.1093/bib/bbaa052 32349124

[36] Driskill JH, Pan D. 2021. The hippo pathway in liver homeostasis and pathophysiology. Annu Rev Pathol 16:299–322. doi:10.1146/annurev-pathol-030420-105050 33234023 PMC8594752

[37] Wu S, Huang J, Dong J, Pan D. 2003. Hippo encodes a Ste-20 family protein kinase that restricts cell proliferation and promotes apoptosis in conjunction with salvador and warts. Cell 114:445–456. doi:10.1016/s0092-8674(03)00549-x 12941273

[38] Huang J, Wu S, Barrera J, Matthews K, Pan D. 2005. The hippo signaling pathway coordinately regulates cell proliferation and apoptosis by inactivating Yorkie, the Drosophila homolog of YAP. Cell 122:421–434. doi:10.1016/j.cell.2005.06.007 16096061

[39] Mo JS, Park HW, Guan KL. 2014. The hippo signaling pathway in stem cell biology and cancer. EMBO Rep 15:642–656. doi:10.15252/embr.201438638 24825474 PMC4197875

[40] Hong AW, Meng Z, Guan KL. 2016. The hippo pathway in intestinal regeneration and disease. Nat Rev Gastroenterol Hepatol 13:324–337. doi:10.1038/nrgastro.2016.59 27147489 PMC5642988

[41] Wang D, Li Z, Li X, Yan C, Yang H, Zhuang T, Wang X, Zang Y, Liu Z, Wang T, Jiang R, Su P, Zhu J, Ding Y. 2022. DUB1 suppresses hippo signaling by modulating TAZ protein expression in gastric cancer. J Exp Clin Cancer Res 41:219. doi:10.1186/s13046-022-02410-5 35820928 PMC9275142

[42] Fang L, Teng H, Wang Y, Liao G, Weng L, Li Y, Wang X, Jin J, Jiao C, Chen L, et al.. 2018. SET1A-mediated Mono-Methylation at K342 regulates YAP activation by blocking its nuclear export and promotes tumorigenesis. Cancer Cell 34:103–118. doi:10.1016/j.ccell.2018.06.002 30008322

[43] Qadir J, Li F, Yang BB. 2022. Circular RNAs modulate hippo-YAP signaling: functional mechanisms in cancer. Theranostics 12:4269–4287. doi:10.7150/thno.71708 35673576 PMC9169354

[44] Higashi T, Hayashi H, Ishimoto T, Takeyama H, Kaida T, Arima K, Taki K, Sakamoto K, Kuroki H, Okabe H, Nitta H, Hashimoto D, Chikamoto A, Beppu T, Baba H. 2015. miR-9-3p plays a tumour-suppressor role by targeting TAZ (WWTR1) in hepatocellular carcinoma cells. Br J Cancer 113:252–258. doi:10.1038/bjc.2015.170 26125451 PMC4506379

[45] Deepitha M, Laura M. 2021. Hepatocellular carcinoma. Nat Rev Dis Primers 7:7. doi:10.1038/s41572-021-00245-6 33479233

[46] Koshiol J, Argirion I, Liu Z, Kim Lam T, O’Brien TR, Yu K, McGlynn KA, Petrick JL, Pinto L, Chen CJ, Hildesheim A, Pfeiffer RM, Lee MH, Yang HI. 2021. Immunologic markers and risk of hepatocellular carcinoma in hepatitis B virus- and hepatitis C virus-infected individuals. Aliment Pharmacol Ther 54:833–842. doi:10.1111/apt.16524 34286851 PMC12332984

[47] Kar A, Samanta A, Mukherjee S, Barik S, Biswas A. 2023. The HBV web: an insight into molecular interactomes between the hepatitis B virus and its host en route to hepatocellular carcinoma. J Med Virol 95:e28436. doi:10.1002/jmv.28436 36573429

[48] Zheng H, Xu B, Fan Y, Tuekprakhon A, Stamataki Z, Wang F. 2025. The role of immune regulation in HBV infection and hepatocellular carcinogenesis. Front Immunol 16:1506526. doi:10.3389/fimmu.2025.1506526 40160817 PMC11949809

[49] Yang L, Zou T, Chen Y, Zhao Y, Wu X, Li M, Du F, Chen Y, Xiao Z, Shen J. 2022. Hepatitis B virus X protein mediated epigenetic alterations in the pathogenesis of hepatocellular carcinoma. Hepatol Int 16:741–754. doi:10.1007/s12072-022-10351-6 35648301

[50] Zhao X, Sun L, Mu T, Yi J, Ma C, Xie H, Liu M, Tang H. 2020. An HBV-encoded miRNA activates innate immunity to restrict HBV replication. J Mol Cell Biol 12:263–276. doi:10.1093/jmcb/mjz104 31865380 PMC7232129

[51] Chavalit T, Nimsamer P, Sirivassanametha K, Anuntakarun S, Saengchoowong S, Tangkijvanich P, Payungporn S. 2020. Hepatitis B virus-encoded MicroRNA (HBV-miR-3) regulates host gene PPM1A related to hepatocellular carcinoma. Microrna 9:232–239. doi:10.2174/2211536608666191104105334 31686644

[52] Li J, Hu ZQ, Yu SY, Mao L, Zhou ZJ, Wang PC, Gong Y, Su S, Zhou J, Fan J, Zhou SL, Huang XW. 2022. CircRPN2 inhibits aerobic glycolysis and metastasis in hepatocellular carcinoma. Cancer Res 82:1055–1069. doi:10.1158/0008-5472.CAN-21-1259 35045986

[53] Sunbul M. 2014. Hepatitis B virus genotypes: global distribution and clinical importance. World J Gastroenterol 20:5427–5434. doi:10.3748/wjg.v20.i18.5427 24833873 PMC4017058

[54] Lin CL, Kao JH. 2017. Natural history of acute and chronic hepatitis B: the role of HBV genotypes and mutants. Best Pract Res Clin Gastroenterol 31:249–255. doi:10.1016/j.bpg.2017.04.010 28774406

[55] Liu B, Yang JX, Yan L, Zhuang H, Li T. 2018. Novel HBV recombinants between genotypes B and C in 3’-terminal reverse transcriptase (RT) sequences are associated with enhanced viral DNA load, higher RT point mutation rates and place of birth among Chinese patients. Infect Genet Evol 57:26–35. doi:10.1016/j.meegid.2017.10.023 29111272

